# Specific premature epigenetic aging of cartilage in osteoarthritis

**DOI:** 10.18632/aging.101053

**Published:** 2016-09-28

**Authors:** Laura Vidal-Bralo, Yolanda Lopez-Golan, Antonio Mera-Varela, Ignacio Rego-Perez, Steve Horvath, Yuhua Zhang, Álvaro del Real, Guangju Zhai, Francisco J Blanco, Jose A. Riancho, Juan J Gomez-Reino, Antonio Gonzalez

**Affiliations:** ^1^ Laboratorio Investigacion 10 and Rheumatology Unit, Instituto Investigacion Sanitaria, Hospital Clinico Universitario de Santiago, Travesia Choupana, sn. 15706- Santiago de Compostela, Spain; ^2^ Grupo de Reumatología, Instituto de Investigación Biomédica de A Coruña, Complexo Hospitalario Universitario de A Coruña, Universidade da Coruña. As Xubias, sn. 15006- A Coruña, Spain; ^3^ Department of Human Genetics, David Geffen School of Medicine, University of California Los Angeles, Los Angeles, CA 90095, USA; ^4^ Discipline of Genetics, Faculty of Medicine, Memorial University of Newfoundland, A1B- St. John's, NL, Canada; ^5^ Department of Internal Medicine, Hospital U. M. Valdecilla-IDIVAL, University of Cantabria, Cardenal Herrera Oria, 39011, Santander, Spain

**Keywords:** osteoarthritis, biological age, epigenetics, DNA methylation, telomere length shortening

## Abstract

Osteoarthritis (OA) is a disease affecting multiple tissues of the joints in the elderly, but most notably articular cartilage. Premature biological aging has been described in this tissue and in blood cells, suggesting a systemic component of premature aging in the pathogenesis of OA. Here, we have explored epigenetic aging in OA at the local (cartilage and bone) and systemic (blood) levels. Two DNA methylation age-measures (DmAM) were used: the multi-tissue age estimator for cartilage and bone; and a blood-specific biomarker for blood. Differences in DmAM between OA patients and controls showed an accelerated aging of 3.7 years in articular cartilage (95 % CI = 1.1 to 6.3, *P* = 0.008) of OA patients. By contrast, no difference in epigenetic aging was observed in bone (0.04 years; 95 % CI = −1.8 to 1.9, *P* = 0.3) and in blood (−0.6 years; 95 % CI = −1.5 to 0.3, *P* = 0.2) between OA patients and controls. Therefore, premature epigenetic aging according to DNA methylation changes was specific of OA cartilage, adding further evidence and insight on premature aging of cartilage as a component of OA pathogenesis that reflects damage and vulnerability.

## INTRODUCTION

OA is the most common chronic disease affecting the joints with about a 45 % lifetime risk of developing OA of the knee [1, 2]. It can affect any joint, but it occurs most often in knees, hips, spine, or hands. Symptoms include pain and stiffness, bony enlargement, crepitus with movement and decreased function of the joint. OA pathogenesis is complex and includes multiple risk factors that are still incompletely known, but old age is a critical contributor [3, 4]. The relationship between old age and OA is not fully understood. Classically, it was suspected that the association was related to the “wear and tear” of articular cartilage by continuous mechanical stress. Today, we know that this model of OA is insufficient because OA involves an active response to injury comprising remodeling of articular cartilage and neighboring bone, in addition of synovial inflammation and damage to ligaments and menisci [2]. In addition, the other component of the association with old age, biological aging, has shown unsuspected complexity, including its multidimensionality, variable progression, possibility of modulation and the pivotal role played by senescent cells [5].

The many facets of biological aging have been typified in nine cellular and molecular hallmarks: genomic instability, telomere attrition, epigenetic alterations, loss of proteostasis, deregulated nutrient sensing, mito-chondrial dysfunction, cellular senescence, stem cell exhaustion, and altered intercellular communication [6]. Variable progression of biological aging with dissociation between biological and chronological age is observed in progeroid syndromes. Less dramatically, it is also observed as a reflection of lifestyle with smoking, heavy drinking, obesity, stress and depression as accelerators, and exercise and caloric restriction as rejuvenators. The pivotal role of cellular senescence and of the senescence-associated secretory phenotype has been established in multiple studies, but most strikingly with the reversal of age-associated changes obtained with their removal [7]. This rejuvenation has been obtained either through genetic manipulation or with senolytic drugs in mice in spite of the eliminated senescent cells were only a minor fraction in mouse tissues [5]. All these aspects could be of relevance for OA as exemplified by the secretory phenotype that includes secretion of metalloproteases and pro-inflammatory mediators, which could be involved in OA cartilage damage [2-4]. There is already persuasive evidence of accelerated biological aging at the affected cartilage [3, 4]. Many of the aging hallmarks have been described as exacerbated in OA chondrocytes and articular cartilage, including telomere length shortening, mitochondrial dysfunction, cellular senescence and genome instability [3, 4]. In contrast, biological age has not been studied in any other joint tissue although a systemic component of premature aging has been suggested by accelerated telomere length shortening in blood cells of 160 hand OA subjects compared with 926 controls [8]. Telomere shortening correlated with radiographic severity of OA in the hands in this study. These findings have not yet been independently confirmed, with a small subsequent study showing no telomere attrition in blood of knee OA patients [9], and a second small study reporting telomere shortening only in knee OA patients experiencing high stress and chronic pain [10]. A systemic premature aging component in OA is an attractive hypothesis because it is congruent with some epidemiological studies that have found increased prevalence of old-age comorbidities [11-14], frailty [15], and mortality in OA patients [16-18]. The two aspects, local and systemic, of premature aging could contribute to OA by further impairing cartilage and joint function, decreasing mobility and increasing joint vulnerability.

An opportunity to explore a different aging hallmark in OA cartilage, bone and blood has become possible thanks to the recent development of biomarkers of epigenetic aging [19-24]. The available biomarkers, called DNA methylation age-measures (DmAM), combine methylation levels at CpG sites that experience methylation changes with aging. The mechanism seems to include slowly accumulating failures of methylation maintenance (epigenetic drift) that could be accelerated by somatic mutations, cell divisions and environmental stress [19, 21, 25, 26]. Some of the changes are tissue-specific; others are shared by several tissues. This motivates a distinction between DmAM that are tissue-specific and include as few as 3 CpG sites showing strong correlation with age in blood, [20, 24] or in saliva [23], and biomarkers applicable to many tissues that require investigating more CpG sites [19, 21]. The most comprehensive is the “epigenetic clock” method by Horvath, which includes 353 CpG and is valid for multiple tissues including bone and cartilage [19, 27, 28]. The DmAM are useful biomarkers of biological age that show accelerated aging in several diseases of old age [19-21, 29, 30] and in subjects under elevated lifetime stress [26], and that correlate with cognitive and physical fitness in the elderly and with all-cause and cause-specific mortality [30-35].

## RESULTS

**Figure 1:**
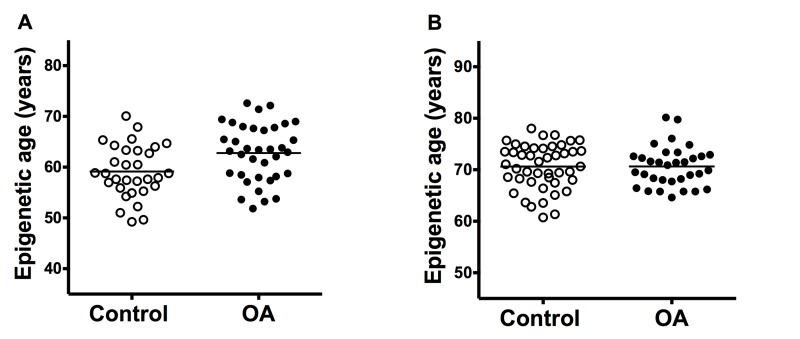
Comparison of epigenetic age in joint tissues from controls and patients with OA (**A**) Accelerated aging in OA cartilage samples (n = 31) in comparison with control cartilage (n = 36) with ΔDmAM = 3.7 years (P = 0.008); and (**B**) no difference (ΔDmAM = 0.04 years, P = 0.3) in bone samples between OA patients (n = 33) and controls (n = 45). Epigenetic ages are represented as age- and sex-adjusted values with horizontal lines for the mean of each group.

The cartilage samples from OA patients showed premature aging in comparison with cartilage from controls (Figure 1A). The difference in the estimated mean age obtained with Horwath's DmAM was of 3.7 years (Table 1). This result was obtained with the whole set of samples that included cartilage from the tibial plateau and from the femoral head. A significant premature aging was also observed with the subgroup of tibial plateau samples, with a mean difference of 5.3 years (95 % CI = 2.4 to 8.2). Cartilages from the femoral heads were too few for meaningful analysis. All the comparisons were adjusted for age and sex as covariates.

**Table 1:** Specific premature epigenetic aging in OA cartilage compared with control cartilage ΔDmAM = (age- and sex-adjusted mean DmAM in OA patients) – (age- and sex-adjusted mean DmAM in controls); CI = confidence interval.

Tissue	OA set	ΔDmAM^a^ (95% CI)	*P*-value
Cartilage	Knee/hip	3.7 (1.1 to 6.3)	0.008
			
Bone	Hip	0.04 (−1.8 to 1.9)	0.3
			
Blood	Hand	0.01 (−1.1 to 1.1)	0.98
	Knee	0.04 (−0.9 to 1.0)	0.9
	Hip	−0.7 (−1.7 to 0.3)	0.11

In contrast with the cartilage results, there were no differences in epigenetic aging of bone (Figure 1B). The mean estimated age obtained from DNA methylation data was very similar in patients with hip OA and in controls (Table 1). The lack of difference was validated in a sub-analysis including only the fracture controls (ΔDmAM = 0.5 years, 95 % CI = −1.50 to 2.54, *P* = 0.6). Bone samples from cadaver controls were too few for meaningful analysis. All the comparisons were adjusted for age and sex as covariates.

**Figure 2:**
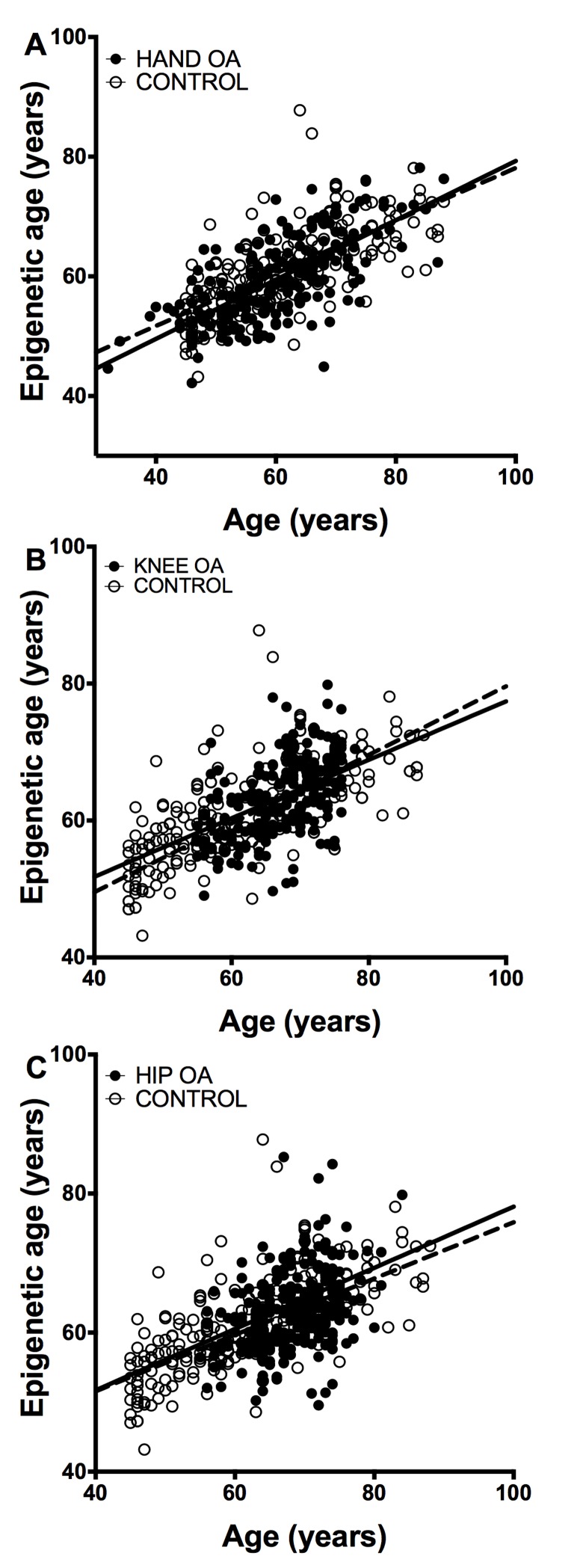
Lack of accelerated epigenetic aging in blood cells of OA patients The scatterplots represent age in the horizontal axis against epigenetic age in the vertical axis from the controls without OA (empty circles, n =182) together with (**A**) the hand OA (n = 206), (**B**) the knee OA (n = 229), and (**C**) the hip OA (n = 273) patients (filled circles). Straight lines represent least squares regression fit to the data.

The study of epigenetic aging in blood required de novo analyzes of methylation levels at the 8 CpG sites. The MS-SNuPE assays showed a 93.0 % call rate, and between-plate CV of 3.2 %. Age of the 182 controls without OA was accurately predicted with the 8CpG DmAM (Figure 2), as shown by the good fit of the mean age estimate (mean difference age – DmAM = −0.1 years, SD = 8.7 years). Comparison of the epigenetic ages obtained in this way did not show differences between OA patients and controls (Figure 2). The epigenetic ages for each of the joint-specific OA subgroups were very similar to the epigenetic age for the control subjects, as shown for the hand OA patients (Figure 2A), knee OA patients (Figure 2B) and hip OA patients (Figure 2C). This similarity in blood cells was clearly shown by the near zero year ΔDmAM (Table 1). The largest difference in blood was observed between patients with hip OA and controls, but it was not significant and with direction opposed to premature aging in the OA subjects. All the comparisons were adjusted for age and sex as covariates.

## DISCUSSION

Our results showed for the first time premature epigenetic aging as detected with DmAM in cartilage of the OA affected joint, but not in bone nearby the OA affected joint, or in blood cells of OA patients irrespective of the joint affected. These results add epigenetic aging to the list of hallmarks of aging showing accelerated changes in OA cartilage. Each of these hallmarks provide complementary and non-redundant evidence of the different facets of the premature biological aging taking place in chondrocytes and the extracellular matrix of the OA affected cartilage. In addition, our results could be interpreted as questioning systemic premature aging in OA, or even a local component of premature aging in nearby bone, but exploration of other aging hallmarks would be required to exclude them.

Previously, several hallmarks of biological aging have been found exacerbated in chondrocytes and cartilage from OA patients [3, 4]. Our work adds epigenetic aging to the list of hallmarks that show premature biological aging in this tissue. This is a significant addition because the different aging hallmarks, although extensively interconnected, show tissue and disease specificity and the involvement of each of them cannot be assumed from the presence of other hallmarks [5, 6, 35]. They need to be tested in the specific tissue or situation under study. This necessity is exemplified by the lack of correlation between epigenetic age and telomere length observed in the elderly population [35]. In addition, diseases of abnormal telomere attrition are different from diseases in which the dominant mechanism is genomic instability and both are different from normal aging. The first group includes pulmonary fibrosis, dyskeratosis congenita and aplastic anemia, whereas genome instability is the dominant aging hallmark in progeroid syndromes such as Hutchinson-Gilford progeria syndrome and Werner's syndrome [5, 6]. In addition, the DNA methylation changes that are included in the Horvath DmAM have been shown to be independent from cellular senescence and mitotic age [19]. Similar lack of redundancy is observed between the other aging hallmarks, making it necessary to study each of them to know their involvement in OA.

Epigenetic changes with age are not restricted to DNA methylation. They encompass also histone modifica-tions regulated by sirtuins and chromatin remodeling [6]. None of the other age-associated epigenetic changes has yet been analyzed in the context of OA, but they are of interest given their potential reversibility as with histone deacetylase inhibitors or inhibitors of histone acetyltransferases as anti-aging drugs [6]. The meaning of these epigenetic changes is still poorly understood. They likely contribute to the loss of transcriptional regulation and increase of transcriptional noise observed with aging [5, 6]. Changes in DNA methylation are concentrated in genes with some functional categories including cell growth and survival, organismal development and cancer [19], and in sites within glucocorticoid response elements [26], but the pattern of hypermethylation and hypomethylation has not yet being linked to specific molecular or cellular processes [19, 20]. Interpretation of the changes should also include the magnitude of the change: the increase in 3.7 years in ΔDmAM observed in the OA cartilage samples of our study is a modest acceleration compared with changes observed in tumoral tissue, but similar to the reported in a recent abstract in hip OA cartilage, which provides independent confirmation of our findings [36], and in blood of HIV infected patients [37], or in blood of Down syndrome patients [29], but larger than the observed in blood from patients with Parkinson disease [38], or in blood of women after menopause [39].

Some of the previously described aging hallmarks are strongest in the damaged cartilage and less clear in cartilage of preserved areas. Hallmarks showing this pattern are mean telomere length shortening [40-42] senescence-associated heterochromatin foci [41, 42], and senescence-associated β-galactosidase (SA-β-gal). These results have been interpreted as representing, at least in part, consequences of cellular stress and the senescence status of the chondrocytes in OA. However, it is possible that epigenetic aging is a biomarker of cellular vulnerability more than of damage and, therefore, a potential target for treatment. Experiments aimed to differentiate between the two mechanisms are necessary but there are already preliminary results showing similar epigenetic aging in damaged and in preserved cartilage from the same OA patient [36]. Potential treatments could include specific senolytic drugs [5] that have not yet been assayed in chondro-cytes, and other approaches with capacity to delay aging in OA chondrocytes as already shown for statins [38] and sirtuin activation [37, 39].

Our results are contrary to widespread premature epigenetic aging given the lack of increased ΔDmAM at the blood and bone levels. However, the only previous direct evidence of a systemic component of accelerate aging in OA was obtained with telomere length in blood cells of OA patients [8, 10], and it is likely that telomere length and DmAM capture different aspects of biological aging [6, 19, 21, 25, 27]. Telomere attrition results from cell divisions, in the absence of the enzyme telomerase, and from DNA damage induced by extrinsic stress, as oxidative or inflammatory stress. The authors that found accelerated telomere length attrition in blood of OA patient interpreted it as reflecting oxidative stress and low-level chronic inflammation [8], or associated with chronic pain and high stress [10]. In contrast, epigenetic aging as measured with DmAM seems to be due to perturbations of the DNA methylation mainte-nance system [19, 21, 25]. Therefore, our results do not question systemic accelerated aging as detected with telomere shortening, but exclude the epigenetic aspect of aging.

The lack of accelerated aging in blood and in bone was not attributable to insufficient power. In effect, blood samples were enough to exclude ΔDmAM half as fast as the observed in cartilage (1-β > 0.95 to exclude a difference of 1.83 years for each of the three joints). Bone samples, in turn, were enough to detect ΔDmAM as large as the observed in cartilage (1-β = 0.90). In addition, the use of different DmAM for cartilage and bone, in one side, and for blood, in the other, does not interfere with our results because no analysis compared results across different DmAM. We also avoided biases due to differences in age or sex between the OA patients and the controls by adjusting for these two variables, as recommended [19-21]. However, limitations of our study are that the different tissues were not from the same subjects, the lack of other joint tissues, and the absence of a larger number of cartilage samples from femoral heads allowing specific analysis of epigenetic aging at this site. The meaning of these limitations seems modest because bone and cartilage are arguably the most relevant tissues in OA [2], and because epigenetic aging in hip cartilage from OA patients has been independently found [36], as already mentioned. In any case, we cannot completely exclude that other tissues or joints show a different behavior than the reporter here, or that additional insight could be gained from analyzing several tissues from the same subjects, as epigenetic age correlation between tissues.

In summary, we have found specific accelerated aging as measured with DNA methylation in cartilage from OA affected joints. Knowledge of the mechanisms of this type of premature aging will help to understand OA pathology, but already it is apparent that these particular mechanisms are not widespread. This was indicated by the results obtained with the same DNA methylation methodology in bone near the affected joint and in blood cells. They showed absence of a systemic component of premature aging. These results cannot exclude that other hallmarks of aging could be more widespread than the DNA methylation changes analyzed here.

## METHODS

### Cartilage and bone epigenetic age

Epigenetic age was estimated with the 353 age-related CpG probes according with Horvath [19]. Methylation information of these sites has been obtained in previous studies addressing cartilage and bone samples (Table 2 and Supplementary Table 1) [43-46]. Cartilage samples were from 31 controls and 36 OA patients (Table 2).

**Table 2:** Main characteristics of the sample collections used in this study N = number of samples, SD = standard deviation.

Tissue	Set	N	Age Mean ± SD (Range)	Woman %
Cartilage	Control knee/hip^a^	31	64.8 ± 15.0 (40-95)	48.4
	Knee/hip OA	36	67.1 ± 9.3 (41-80)	75.0
				
Bone	Control hip^a^	45	78.0 ± 11.0 (40-104)	93.3
	Hip OA	33	75.4 ± 6.7 (58-89)	100.0
				
Blood	Control	182	60.7 ± 11.5 (45-88)	46.7
	Hand OA	206	60.6 ± 10.1 (32-88)	88.4
	Knee OA	229	67.7 ± 5.6 (55-78)	82.1
	Hip OA	273	68.4 ± 5.5 (55-84)	59.7

a 3 cartilage and 4 bone control samples were from undefined localization

The controls were from tibial plateau of 18 cadavers with no macroscopic signs of OA [43], and the femoral head of 10 subjects with hip fracture and without macroscopic or microscopic evidence of OA [46]. In addition, 3 cartilage samples from cadavers without information of status and location were included [45]. The OA samples included 29 from the tibial plateau of severe knee OA patients [43, 46], and 7 from the femoral head of severe hip OA patients [46], obtained at the time of joint replacement. Bone samples were from 45 controls and 33 hip OA patients (Table 2). The controls included femoral heads of 34 subjects with osteoporotic hip fracture (OP) and 7 cadavers [44]. They lacked OA lesions on macroscopic examination of the hip joints and the bone pieces excluded subchondral and fractured regions. Patients with fractures due to high-energy trauma or with disorders causing secondary OP or OA were not included. In addition, 4 control bone samples from cadavers that lacked detailed information of status and place of retrieval were included.[45] The bone samples of the 33 hip OA patients were obtained from femoral heads at the time of total joint replacement for primary hip OA [44]. Methylation data were obtained either with the Human Methylation 27 BeadChip (Illumina), [43, 44] or with the HumanMethylation 450 Bead-Chip microarray (Illumina, San Diego, California, USA) [45, 46]. These samples were obtained with informed consent of the donors and approval of the relevant ethics committees as reported in the primary publications [43-46].

### Analysis of epigenetic aging in blood

Epigenetic aging in blood was assessed with a 8 CpG DmAM specific for whole blood and amenable to assay in large number of samples [24]. Methylation data were obtained for this study with methylation-sensitive single-nucleotide primer extension (MS-SNuPE) following the reported procedure [47]. Genomic DNA from 890 subjects of Spanish ancestry was assayed (Table 2 and Supplementary Table 1). This collection of samples included 182 controls recruited at the time of intravenous urography. They had not OA signs at exploration including both hands or in the radiographs either at the hip or column joints, and they did not complain of OA symptoms in a systematic question-naire. The remaining 708 subjects were suffering from primary OA as assessed by a rheumatologist. The subjects affected by knee OA, 229, or hip OA, 273, were selected from consecutive patients aged 55–75 years at the time of surgery that were undergoing total joint replacement. The patients with hand OA, 206, were selected among those attending the Rheumatology Unit fulfilling the American College of Rheumatology classification criteria for hand OA [48]. Exclusion criteria were inflammatory, infectious, traumatic or congenic joint pathology, as well as, lesions due to crystal deposition or osteonecrosis. Morbid obesity and occupational strain were not exclusion causes. All donors provided blood DNA samples for genetic studies with written informed consent according to the Declaration of Helsinki (most recently at the General Assembly on October 2008) and the approval of the Ethics Committee for Clinical Research of Galicia, as described [49].

### Statistical analysis

We estimated the epigenetic age of the cartilage and bone samples with Horvath's DmAM [19], and of the blood samples with the 8 CpG DmAM [24]. Comparisons between samples from OA patients and controls were done with analysis of variance (ANOVA) including age and sex as covariates. Mean differences in DmAM estimates (ΔDmAM) were calculated as:

ΔDmAM = (age- and sex-adjusted mean DmAM in OA patients) – (age- and sex-adjusted mean DmAM in controls)

Age- and sex-adjustment was done with the residuals from multiple linear regression of estimated age *versus* age and sex. All these analyses were done with Statistica 7.0 (Stat Soft, Inc.). Post-hoc power analysis was done with G*Power 3 for α = 0.05 [50].

## SUPPLEMENTARY MATERIAL TABLE


