# Supplementary material for: Specific premature epigenetic aging of cartilage in osteoarthritis
Source: Aging (Albany NY). 2016 Sep 28;8(9):2222–30. doi: 10.18632/aging.101053 (PMC5076459; doi:10.18632/aging.101053)
Supplement: Supplementary file 1 [file aging-08-2222-s001.pdf]

## SUPPLEMENTARY MATERIAL

**Table S1. Detailed description of the sample collections used in this study. N= Sample size; Std Dev = Standard deviation**

| Tissue | Study | Set | N | Age Mean | Age Range | Std.Dev. | Woman % |
|---|---|---|---|---|---|---|---|
| Blood | Current study | Controls | 182 | 60.70 | 45 to 88 | 11.51 | 46.70 |
| | | Hand OA | 206 | 60.58 | 32 to 88 | 10.06 | 88.35 |
| | | Knee OA | 229 | 67.66 | 55 to 78 | 5.63 | 82.09 |
| | | Hip OA | 273 | 68.38 | 55 to 84 | 5.50 | 59.70 |
| Cartilage | Fernández-Tajes J et al, Ann Rheum Dis 2014; 73:668 | Knee Controls | 18 | 59.28 | 40 to 79 | 10.83 | 33.33 |
| | | Knee OA | 29 | 68.52 | 54 to 79 | 7.25 | 69.00 |
| | Aref-Eshghi E et al, BMC Musculoskelet Disord 2015; 16:287 | Knee OA | 6 | 65.35 | 54 to 78 | 10.63 | 100.00 |
| | | Hip Controls | 10 | 79.37 | 63 to 95 | 11.38 | 90.00 |
| | | Hip OA | 7 | 60.93 | 41 to 80 | 14.29 | 100.00 |
| | Lokk K et al, Genome Biol 2014; 15:R54 | Controls | 3 | 49.00 | 40 to 54 | 7.81 | 0.00 |
| Bone | | Controls | 4 | 51.75 | 40 to 60 | 8.42 | 25.00 |
| | Delgado-Calle J et al, Arthritis Rheum 2013; 65:197 | Hip Cadaver | 7 | 80.29 | 69 to 92 | 8.08 | 100.00 |
| | | Hip Fracture | 34 | 80.68 | 65 to 104 | 7.11 | 100.00 |
| | | Hip OA | 33 | 75.42 | 58 to 89 | 6.74 | 100.00 |
